# Effects of preconditioning with heat stress on acute exercise‐induced intracellular signaling in male rat gastrocnemius muscle

**DOI:** 10.14814/phy2.15913

**Published:** 2024-01-07

**Authors:** Toshinori Yoshihara, Shohei Dobashi, Hisashi Naito

**Affiliations:** ^1^ Graduate School of Health and Sports Science Juntendo University Chiba Japan; ^2^ Institute of Health and Sport Sciences University of Tsukuba Ibaraki Japan

**Keywords:** extracellular signal‐related kinase, heat shock protein 72, mechanistic target of rapamycin signaling, protein kinase B/Akt

## Abstract

Heat stress (HS) induces Akt/mTOR phosphorylation and FoxO3a signaling; however, whether a prior increase in heat shock protein 72 (HSP72) expression affects intracellular signaling following eccentric exercise remains unclear. We analyzed the effects of HS pretreatment on intramuscular signaling in response to acute exercise in 10‐week‐old male Wistar rats (*n* = 24). One leg of each rat was exposed to HS and the other served as an internal control (CT). Post‐HS, rats were either rested or subjected to downhill treadmill running. Intramuscular signaling responses in the red and white regions of the gastrocnemius muscle were analyzed before, immediately after, or 1 h after exercise (*n* = 8/group). HS significantly increased HSP72 levels in both deep red and superficial white regions. Although HS did not affect exercise‐induced mTOR signaling (S6K1/ERK) responses in the red region, mTOR phosphorylation in the white region was significantly higher in CT legs than in HS legs after exercise. Thr308 phosphorylation of Akt showed region‐specific alteration with a decrease in the red region and an increase in the white region immediately after downhill running. Overall, a prior increase in HSP72 expression elicits fiber type‐specific changes in exercise‐induced Akt and mTOR phosphorylation in rat gastrocnemius muscle.

## 1 INTRODUCTION

Various stimuli, such as mechanical stress, physical activity, nutrients, and growth factors, affect skeletal muscle adaptation (Rommel et al., 2001; Sakamoto et al., 2002; Vary & Lynch, 2006). These stimuli act as triggers that regulate protein synthesis and breakdown to maintain skeletal muscle mass. Growing evidence has demonstrated that heat stress (HS) leads to increased muscle protein content and muscle hypertrophy (Goto et al., 2003; Kobayashi et al., 2005; Uehara et al., 2004). Although the specific mechanism underlying HS‐induced skeletal muscle hypertrophy has not been clarified, heat shock protein (HSP) plays a crucial role as a molecular chaperone in facilitating protein translation (Craig, 1993; Goto et al., 2003; Naito et al., 2000; Nelson et al., 1992). Moreover, our previous study showed that thermal stress induces the phosphorylation of the mechanistic target of rapamycin (mTOR) signaling molecules, which are involved in muscle protein synthesis, in a temperature‐dependent manner (Yoshihara et al., 2013), suggesting that such direct (activation of the mTOR signaling pathway) and/or indirect (translation assistance by HSPs) effects contribute to thermal stress‐induced muscle hypertrophy.

A previous study demonstrated that HS preconditioning or the subsequent accumulation of HSPs inhibits tenotomy‐induced hypertrophy in the plantaris muscle in rats (Frier & Locke, 2007). This study used a rat model of compensatory hypertrophy of the plantaris muscle induced by cooperative tendonectomy and reported that overload‐induced muscle hypertrophy is suppressed even though HSP72 expression is increased by prior application of heat at 42°C for 15 min. This finding indicates that HS pretreatment has adverse effects on muscle adaptation to exercise, but the role of HSP72 in activating intracellular signaling involved in muscle hypertrophy remains unclear. Moreover, as a rat model of compensatory hypertrophy cannot be physiologically stimulated, the compensatory hypertrophy resulting from surgical removal, which yields a constant overload, is believed to demonstrate the remarkable gain of skeletal muscle mass. However, it is not directly applicable to resistance training in humans (Schmoll et al., 2018). In contrast, eccentric muscle contractions appear to be a more effective model to confirm the role of HSP72 in activating intracellular signaling involved in muscle hypertrophy under physiological conditions. Eccentric exercise causes muscle damage to induce muscle hypertrophy in rodent skeletal muscles (Armstrong et al., 1983; Kano et al., 2004; Ogilvie et al., 1988; Tsumiyama et al., 2014). Moreover, downhill running (DR) induced the upregulation of mitogen‐activated protein kinases in mice gastrocnemius‐soleus complexes (Boppart et al., 2006). We have previously demonstrated that a bout of DR (incline: −16°, speed: 16 m/min) for 10 min induced mTOR/S6K1/S6 phosphorylation (Chang et al., 2022; Yoshihara et al., 2019), and repeated bouts of the exercise‐induced skeletal muscle hypertrophy partially via mTOR signaling in male Wistar rats (Chang et al., 2022; Yoshihara et al., 2019). Given that excessive overload from eccentric exercise (DR) inhibits hypertrophy in mice skeletal muscles (Rocha et al., 2016), it is necessary to use an eccentric exercise model under physiological conditions in vivo to investigate the effects of HS pretreatment on intracellular signaling. Therefore, the aim of this study was to clarify the effects of HS pretreatment on intracellular signaling in muscle cells following transient eccentric downhill exercise in order to stimulate protein synthesis‐related signaling (e.g., mTOR singling pathway).

## 2 MATERIALS AND METHODS

### 2.1 Experimental animals and heat treatment

This study was approved by the Juntendo University Animal Care Committee (Approval No. 2021‐31) and followed the guiding principles for the care and use of laboratory animals set forth by the Physiological Society of Japan. Herein, we used 24 male Wistar rats, which were housed in a climate‐controlled room (23 ± 1°C, 55 ± 5% relative humidity, and 12:12‐h light–dark photoperiod) and fed standard rat chow and water ad libitum. As estrogen is a factor affecting postexercise HSP 72 level (Paroo et al., 1999), we used male rats in the present study.

Following acclimatization, at 10 weeks of age, one leg of each rat was immersed in 43°C water for 30 min (heat‐stressed leg, HS) under anesthesia with pentobarbital sodium (60 mg/kg) as described previously (Yoshihara et al., 2016). To control intra‐subject variation in the response to local HS, the other leg served as an internal control (control leg, CT). HS was applied twice at a 48‐h interval to ensure an increase in HSP72 expression in both deep red and superficial white regions of the muscles based on previous studies (Oishi et al., 2003; Yoshihara et al., 2016). The time interval of repeated HS was selected based on the results of a previous study, which showed that HSP72 expression peaks between 24 and 48 h following HS (Selsby & Dodd, 2005). The rectal temperature of rats increased from 37.2 ± 0.4°C to 39.1 ± 0.3°C (Δ2.0°C) during the first HS and 36.7 ± 0.3°C to 38.8 ± 0.4°C (Δ2.1°C) during the second HS. Thereafter, the rats were randomly assigned into two groups: non‐exercised sedentary control (*n* = 8) and acute exercise (*n* = 16) groups.

### 2.2 Acute treadmill exercise and blood sampling

**FIGURE 1:**
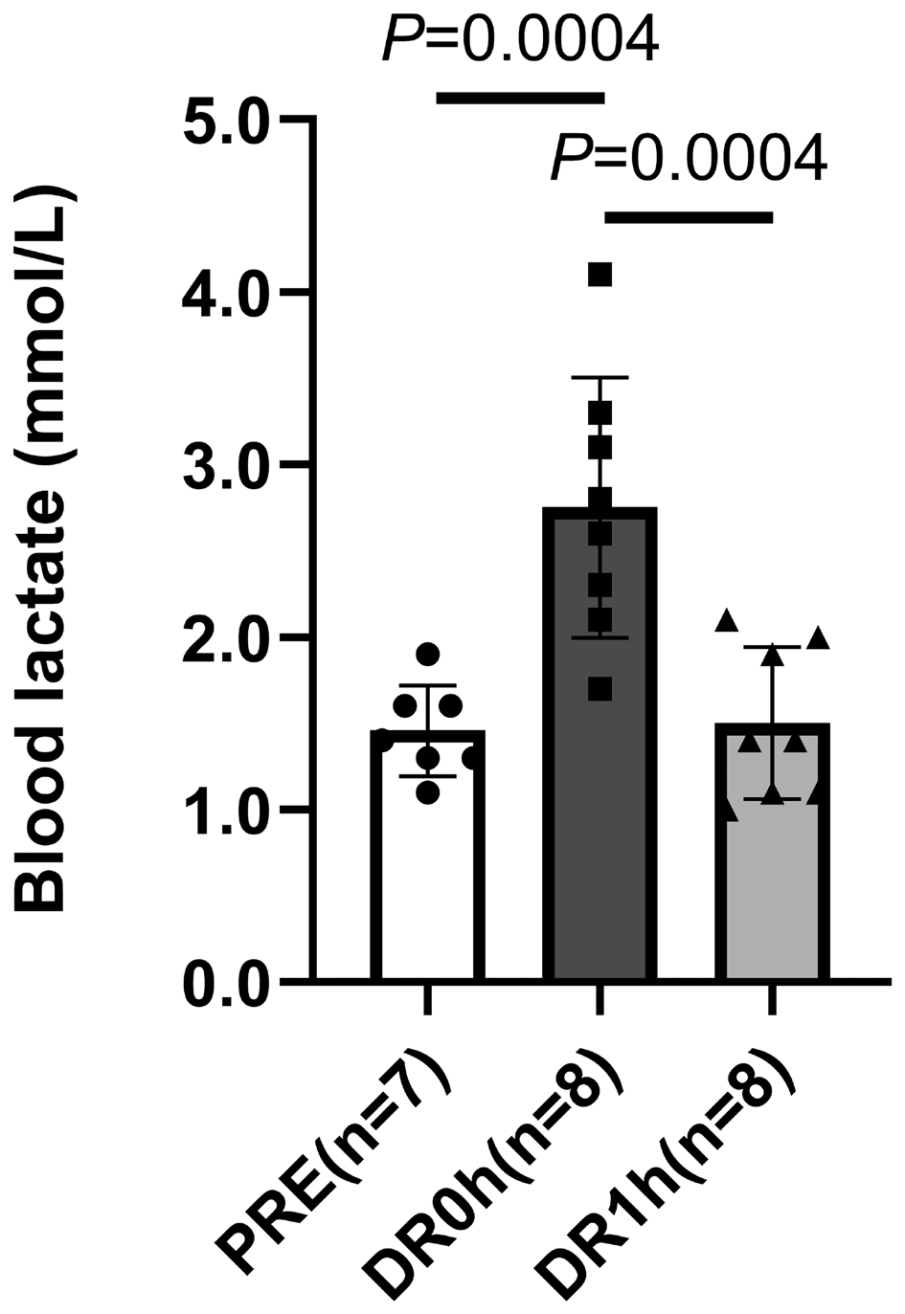
Blood lactate concentration after acute treadmill exercise. Blood samples were collected before exercise (SED, *n* = 7), immediately after exercise (DR0h, *n* = 8), and 1 h after exercise (DR1h, *n* = 8). The data are expressed as mean ± standard deviation (SD).

Forty‐eight hours after the second HS, the rats in the acute exercise groups were subjected to a bout of DR for 10 min (16 m/min, −16% incline) as described previously (Yoshihara et al., 2019); this approach has been used to develop a physiologically relevant model of eccentric loading for inducing muscle hypertrophy (Armstrong et al., 1983; Kano et al., 2004; Ogilvie et al., 1988). Blood samples were collected before exercise (SED, *n* = 7), immediately after exercise (DR0h, *n* = 8), and 1 h after exercise (DR1h, *n* = 8). The blood lactate concentration was significantly elevated to 2.8 mmol/L immediately after exercise (SED, 1.5 ± 0.3; DR0h, 2.8 ± 0.8; DR1h, 1.5 ± 0.4, *p* < 0.05, Figure 1).

### 2.3 Muscle protein synthesis and muscle sampling

We used the in vivo surface sensing of translation (IV‐SUnSET) method to evaluate muscle protein synthesis in the gastrocnemius muscles (Ogasawara et al., 2017). Briefly, puromycin (0.04 μmol/g body weight) was diluted in 0.02 M phosphate‐buffered saline and administered to anesthetized rats in the SED and DR1h groups via intraperitoneal (IP) injection, for 15 min after which, the gastrocnemius muscles were collected. Western blotting was performed and all protein ladder bands were detected using puromycin antibodies.

The gastrocnemius muscle samples (red and white regions of the muscle samples, separately) of both legs (control and heat‐stressed) were collected before, immediately after, and 1 h after exercise (SED [*n* = 8], DR0h [*n* = 8], and DR1h [*n* = 8], respectively) and rapidly frozen in liquid nitrogen.

### 2.4 Muscle preparation

Frozen red and white parts of gastrocnemius muscle tissues were powdered, and 20–30 mg of tissue were homogenized in 10 volumes of ice‐cold homogenization buffer: 20 mM HEPES pH 7.4, 4 mM EGTA, 0.1 mM EDTA, 10 mM MgCl_2_, and 1% Triton X‐100, containing complete EDTA‐free protease (Roche, Penzberg, Germany) and PhosSTOP phosphatase (Roche) inhibitor cocktails, as previously described (Yoshihara et al., 2016). The lysate was then centrifuged at 12,000 × ***g*** for 15 min at 4°C and the supernatant was collected. Protein concentration was determined using a bicinchoninic acid protein assay kit (Thermo Fisher Scientific, Waltham, MA, USA).

### 2.5 Sodium dodecyl sulfate–polyacrylamide gel electrophoresis, western blotting, and immunodetection

Ten micrograms of total protein from each sample were loaded onto precast 4%–15% Tris‐glycine extended polyacrylamide gels (Bio‐Rad, Copenhagen, Denmark), and the proteins were then transferred to polyvinylidene difluoride membranes (Bio‐Rad). The membranes were blocked in a blocking buffer (EveryBlot; Bio‐Rad) for 20 min. After several washes, the membranes were incubated overnight with primary antibodies against the following proteins: phosphorylated Ser473‐Akt (1:2000; catalog #4060), phosphorylated Thr308‐Akt (1:2000; #2965), Akt (1:2000; #2920), phosphorylated Ser2448‐mTOR (1:2000; #2971), mTOR (1:2000; Cell Signaling, #2983), phosphorylated Thr389‐p70S6 kinase (1:2000; #9234), p70S6K (1:2000; #9202), phosphorylated Thr37/46‐4E‐BP1 (1:2000; #2855), and 4E‐BP1 (1:2000; #9452), phosphorylated Thr202/Tyr204 ERK1/2 (1:2000; #4370), ERK (1:2000; #4695), phosphorylated Thr180/Tyr182 p38 MAPK (1:2000; #4511), p38 MAPK (1:2000; #8690), phosphorylated Ser253‐FoxO3a (1:2000; #13129), c‐Myc (1:2000; #9402), and LC3A/B (1:2000; #4108) (all from Cell Signaling Technology, Beverly, MA, USA); FoxO3a (1:2000; 04‐1007) and Puromycin (1:2000; MABE343) (from Millipore, Temecula, CA, USA); and 4‐hydroxynonenal (4‐HNE) (1:2000; ab46545, Abcam, Cambridge, MA, USA). After several washes, the membranes were incubated with anti‐rabbit or ‐mouse horseradish peroxidase‐conjugated secondary antibodies (1:10000; #7074 and #7076, Cell Signaling Technology) in dilution buffer (3% bovine serum albumin/Tris‐buffered saline with Tween 20 (TBS‐T)) for 1 h at room temperature (25–26°C). After washing three times, protein bands were visualized using ECL Prime reagent (Amersham, Piscataway, NJ, USA), and the signal was recorded using a ChemiDoc Touch imaging system (Bio‐Rad). Signal intensity was analyzed using Image Lab v.5.2.1 software (Bio‐Rad). Revert 700 Total Protein Stain (LI‐COR Biosciences, Lincoln, NE, USA) was used as a loading control. To analyze multiple proteins on the same membrane, membranes were washed with Restore Western Blot Stripping Buffer (Thermo Scientific) according to the manufacturer's protocol. Then, the blots were washed and blocked, before the blot was reproved with other primary antibodies.

To analyze HSP expression, membranes were incubated with anti‐HSP70/72 alkaline phosphatase conjugate (1:1000; Stressgen, SPA‐810AP) and anti‐heat shock cognate (HSC) 73 alkaline phosphatase conjugate (1:2000; Stressgen, SPA‐815AP) in dilution buffer for 1 h at room temperature (25–26°C). The membranes were subsequently washed in TBS‐T, and the signal was recorded as described above.

### 2.6 Myosin heavy chain composition

Changes in muscle fiber type of SED rats were assessed based on myosin heavy chain (MHC) composition using a modified version (Sugiura & Murakami, 1990) of a previously described method (Ogura et al., 2011). Gels were scanned using the ChemiDoc Touch Imaging System (Bio‐Rad) and the relative percentages of MHC isoforms were analyzed using Image Lab v.5.2.1 software (Bio‐Rad). In this study, we could not consistently separate the fast MHC IIa type from the fast MHC IId/x type because of some technical issues; therefore, we regarded the two protein bands as MHC IIa/x in the deep red region of the gastrocnemius muscle (Lochynski et al., 2013). Nonetheless, the percentages of IIa and IIx have been reported as references.

### 2.7 Statistical analysis

Values are expressed as mean ± standard deviation of the mean. Statistical significance was evaluated by two‐way (heat stress × exercise) analysis of variance at each endpoint (immediately and 1 h after downhill exercise). Simple effects tests were performed when the interaction was significant. When significant main effects were found (without significant interaction), pairwise comparisons were performed where necessary using Sidak's method. Unpaired Student's *t*‐test was used to compare variables between the CT and HS legs within each endpoint. The correlation between the HSP72 level and intracellular signaling was analyzed using simple linear regression and Pearson's correlation analysis. Results with *p* < 0.05 were considered statistically significant. All statistical analyses were performed using Prism v.8.0 software (GraphPad Inc., La Jolla, CA, USA).

## 3 RESULTS

### 3.1 MHC composition

In the red region of the muscle, there were no significant differences in the percentage of Type I, IIa/x, and IIb MHC between the CT (Type I, 36.4% ± 4.2%; IIa/x, 53.5% ± 3.6% (ref. IIa, 16.6% ± 4.6%; IIa/x, 36.9% ± 6.3%); IIb, 10.1% ± 5.8%) and HS legs (Type I, 35.3% ± 8.5%; IIa/x, 52.0% ± 4.8% (ref. IIa, 19.6% ± 4.7%; IIa/x, 32.4% ± 4.1%); IIb, 12.7% ± 8.0%) of SED rats. In addition, there were no significant differences in the percentage of Type IIx and IIb MHC in the white region of the muscle between the CT (IIx, 38.8% ± 6.0%; IIb, 61.2% ± 6.0%) and HS legs (IIx, 42.0% ± 6.8%; IIb, 58.0% ± 6.8%) of SED rats.

### 3.2 HSP72 and HSC73 expression

**FIGURE 2:**
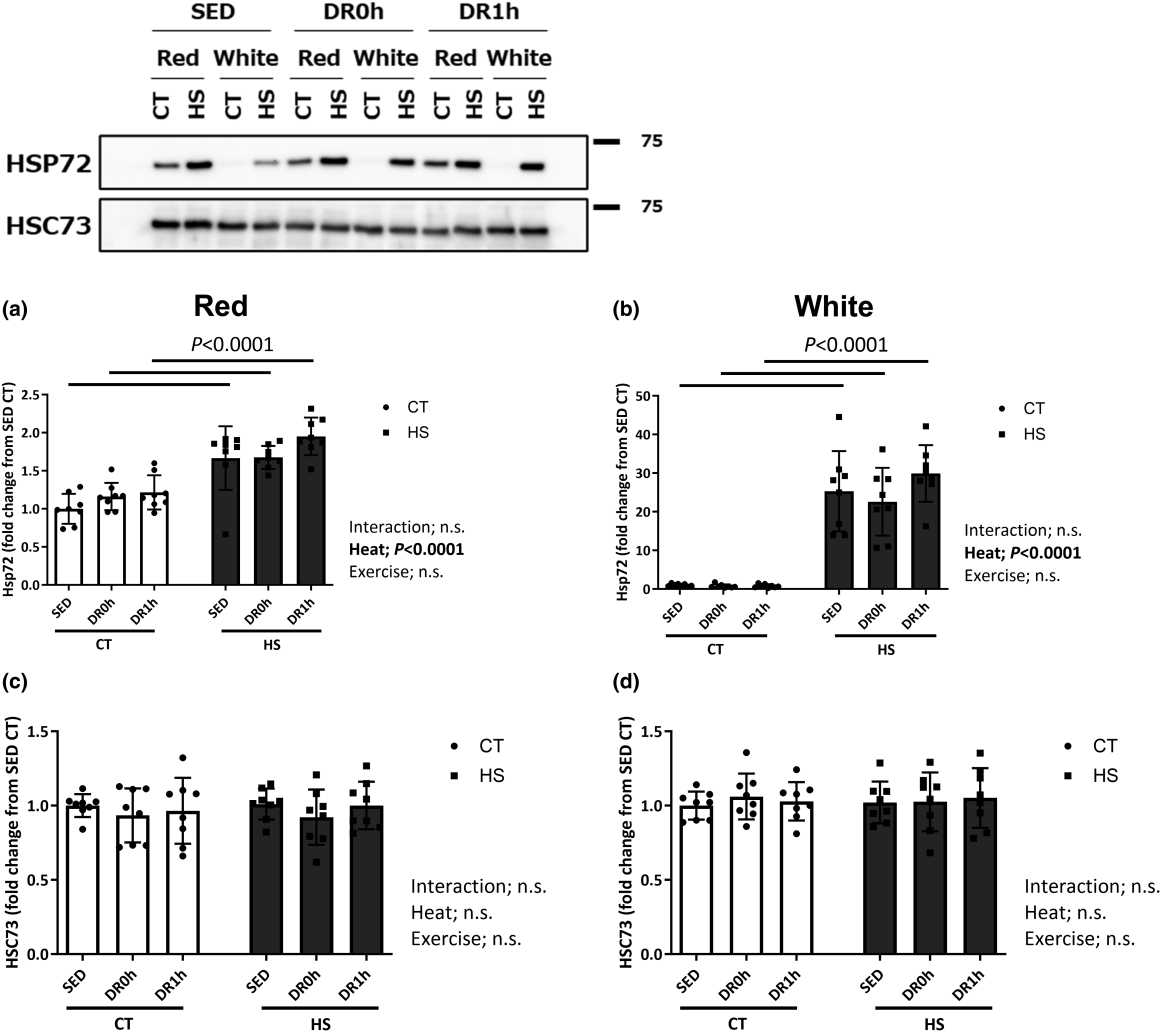
Expression of HSP72 and HSC73. Protein expression of HSP72 (a, b) and HSC73 (c, d) in the red and white regions of the gastrocnemius muscle following downhill exercise and representative blots. After detection of SP72, the blot was reproved for HSC73. CT, control leg; HS, heat‐stressed leg. Samples were collected before exercise (SED) and immediately (DR0h) and 1 h (DR1h) after exercise. The average SED value of the CT leg is presented as 1.0 and the remaining conditions are normalized to this condition. The data are expressed as mean ± standard deviation (SD); *n* = 8 per time point. The results of two‐way ANOVA are displayed.

Figure 2 shows the expression of HSP72 and HSC73 in the red and white regions of the muscles. The HSP72 level was significantly altered by HS pretreatment in both red and white regions (*p* < 0.0001 and 0.0001, respectively; Figure 2a,b), and there were significant differences between the CT and HS legs in the red and white regions of the muscle. No significant changes were observed in the levels of HSC73 in either the red or white region of the muscle (Figure 2c,d).

### 3.3 mTOR and downstream signaling

**FIGURE 3:**
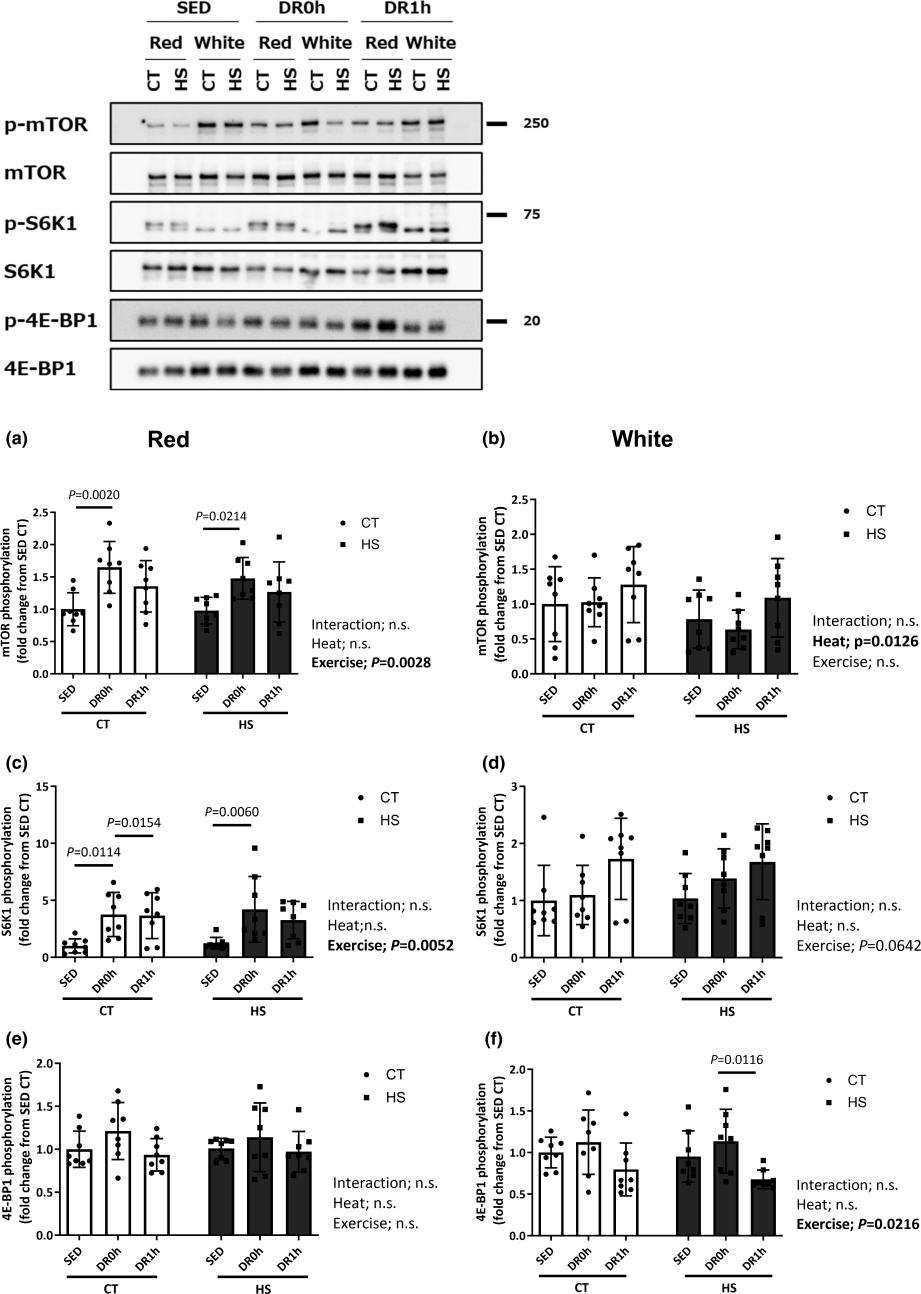
Phosphorylation of mTOR and its downstream signaling intermediates. Phosphorylation ratio of mTOR (a, b), S6K1 (c, d), and 4E‐BP1 (e, f) in the deep red and white regions of the gastrocnemius muscle following downhill exercise and representative blots. After the detection of phospho‐proteins, the blots were reproved for their total proteins. CT, control leg; HS, heat‐stressed leg. Samples were collected before exercise (SED) and immediately (DR0h) and 1 h (DR1h) after exercise. The average SED value of the CT leg is presented as 1.0 and the remaining conditions are normalized to this condition. The data are expressed as mean ± standard deviation (SD); *n* = 8 per time point. The results of two‐way ANOVA are displayed.

Figure 3 shows the observed changes in the expression and phosphorylation levels of mTOR (Ser2448), 70‐kDa ribosomal protein S6 kinase 1 (S6K1) (Thr389), and initiation factor (eIF) 4E‐binding protein‐1 (4E‐BP1) (Thr37/46) after exercise in the red and white regions of the muscle. The expression of mTOR, S6K1, and 4E‐BP1 was not significantly different in the red and white regions of either experimental group (data not shown). In the red region, the Ser2448 phosphorylation level of mTOR significantly increased upon exercise (*p* = 0.0034; Figure 3a); however, this change was unaffected by HS. In contrast, the mTOR phosphorylation level significantly decreased in response to HS in the white region of the muscle (*p* = 0.0034; Figure 3b). Similarly, although the Thr389 phosphorylation level of S6K1 significantly changed in the red region (*p* = 0.0052; Figure 3c) and tended to increase in the white region after downhill exercise (*p* = 0.0642; Figure 3d), these changes were unaffected by HS. No significant change in the Thr37/46 phosphorylation level of 4E‐BP1 was observed in the red region (Figure 3e), whereas a significant change in 4E‐BP1 phosphorylation was observed after downhill exercise in the white region of the muscle (*p* = 0.0216; Figure 3f).

### 3.4 Mitogen‐activated protein kinase (ERK and p38) phosphorylation

**FIGURE 4:**
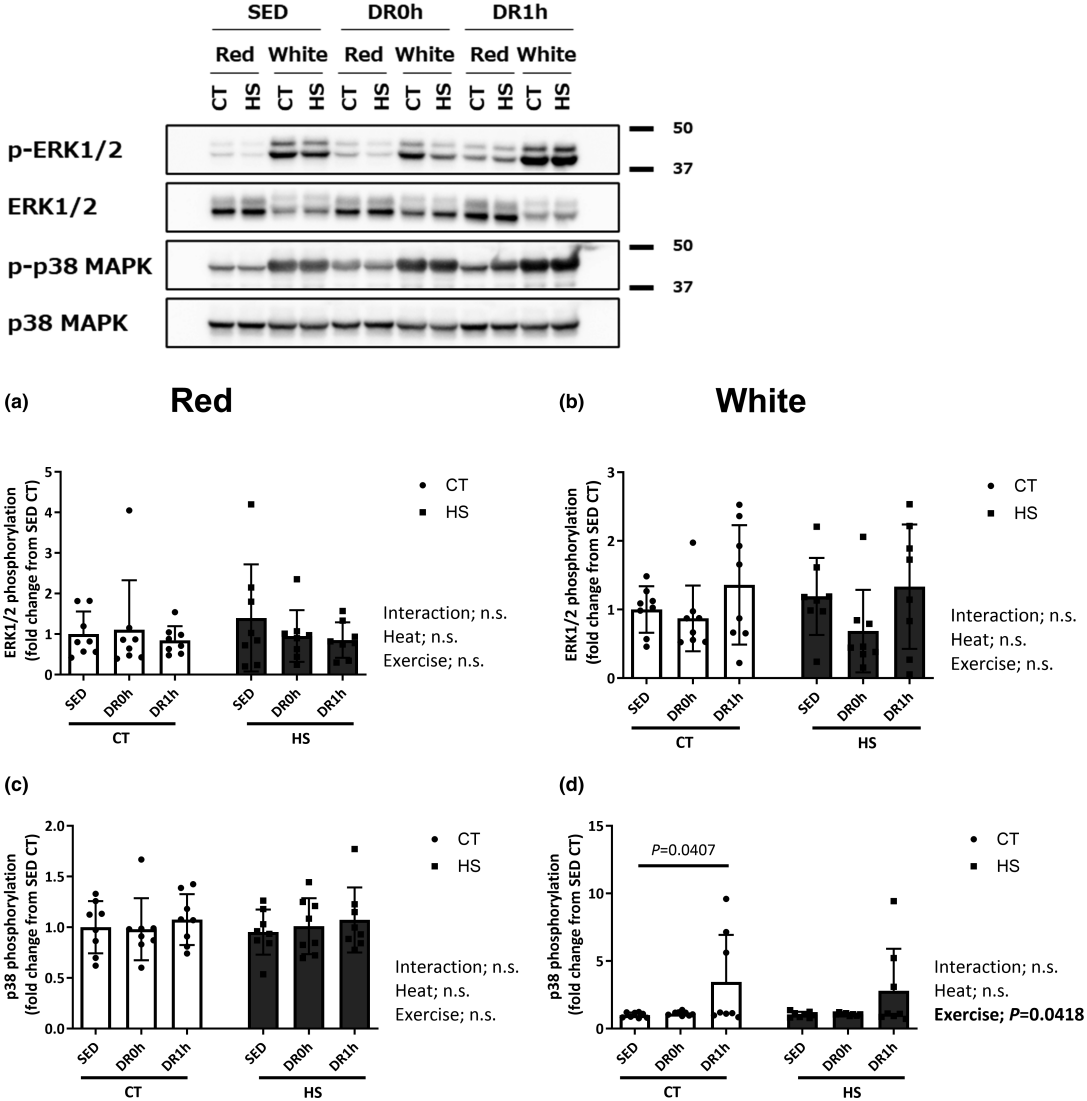
Mitogen‐activated protein kinase (ERK and p38) phosphorylation. Phosphorylation ratio of p44/42 MAPK (Erk1/2) (Thr202/Tyr204) (a, b) and p38 MAPK (Thr180/Tyr182) (c, d) in the red and white regions of the gastrocnemius muscle following downhill exercise and representative blots. After the detection of phospho‐proteins, the blots were reproved for their total proteins. CT, control leg; HS, heat‐stressed leg. Samples were collected before exercise (SED) and immediately (DR0h) and 1 h (DR1h) after exercise. The average SED value of the CT leg is presented as 1.0 and the remaining conditions are normalized to this condition. The data are expressed as mean ± standard deviation (SD); *n* = 8 per time point. The results of two‐way ANOVA are displayed.

Figure 4 depicts the phosphorylation ratio of extracellular signal‐regulated kinase (ERK) (Thr202/Tyr204) and p38 MAPK (Thr180/Tyr182) in the red and white regions of the muscle. The total protein expression of ERK and p38 MAPK was not significantly different among the experimental groups in either region of the muscle (data not shown). No significant changes were observed in ERK phosphorylation levels at Thr202/Tyr204 in both red and white regions of the muscles (Figure 4a,b). A significant increase in p38 MAPK phosphorylation level at Thr180/Tyr182 was observed after downhill exercise in the white region of the muscle (Figure 4d) but this change was unaffected by HS.

### 3.5 c‐Myc protein expression

**FIGURE 5:**
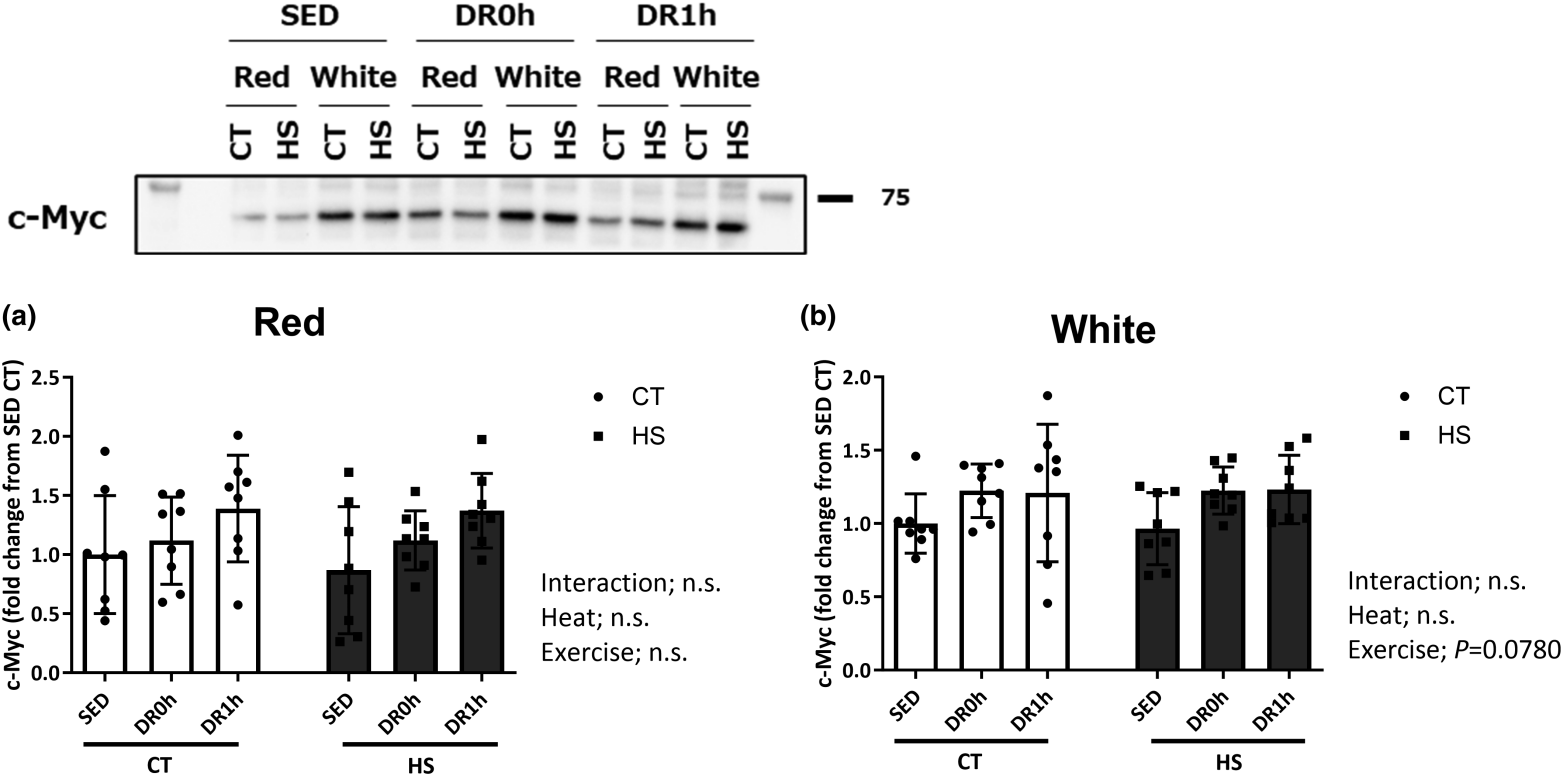
c‐Myc protein expression. Protein expression of c‐Myc in the red (a) and white (b) regions of the gastrocnemius muscle following downhill exercise and representative blots. CT, control leg; HS, heat‐stressed leg. Samples were collected before exercise (SED) and immediately (DR0h) and 1 h (DR1h) after exercise. The average SED value of the CT leg is presented as 1.0 and the remaining conditions are normalized to this condition. The data are expressed as mean ± standard deviation (SD); *n* = 8 per time point. The results of two‐way ANOVA are displayed.

Figure 5 presents c‐Myc expression levels in the red and white regions of the muscle. No significant change was observed in the c‐Myc level in the red region (Figure 5a), although the c‐Myc level in the white region of the muscle tended to increase after downhill exercise (*p* = 0.0780; Figure 5b).

### 3.6 Akt (Ser473 and Thr308) phosphorylation

**FIGURE 6:**
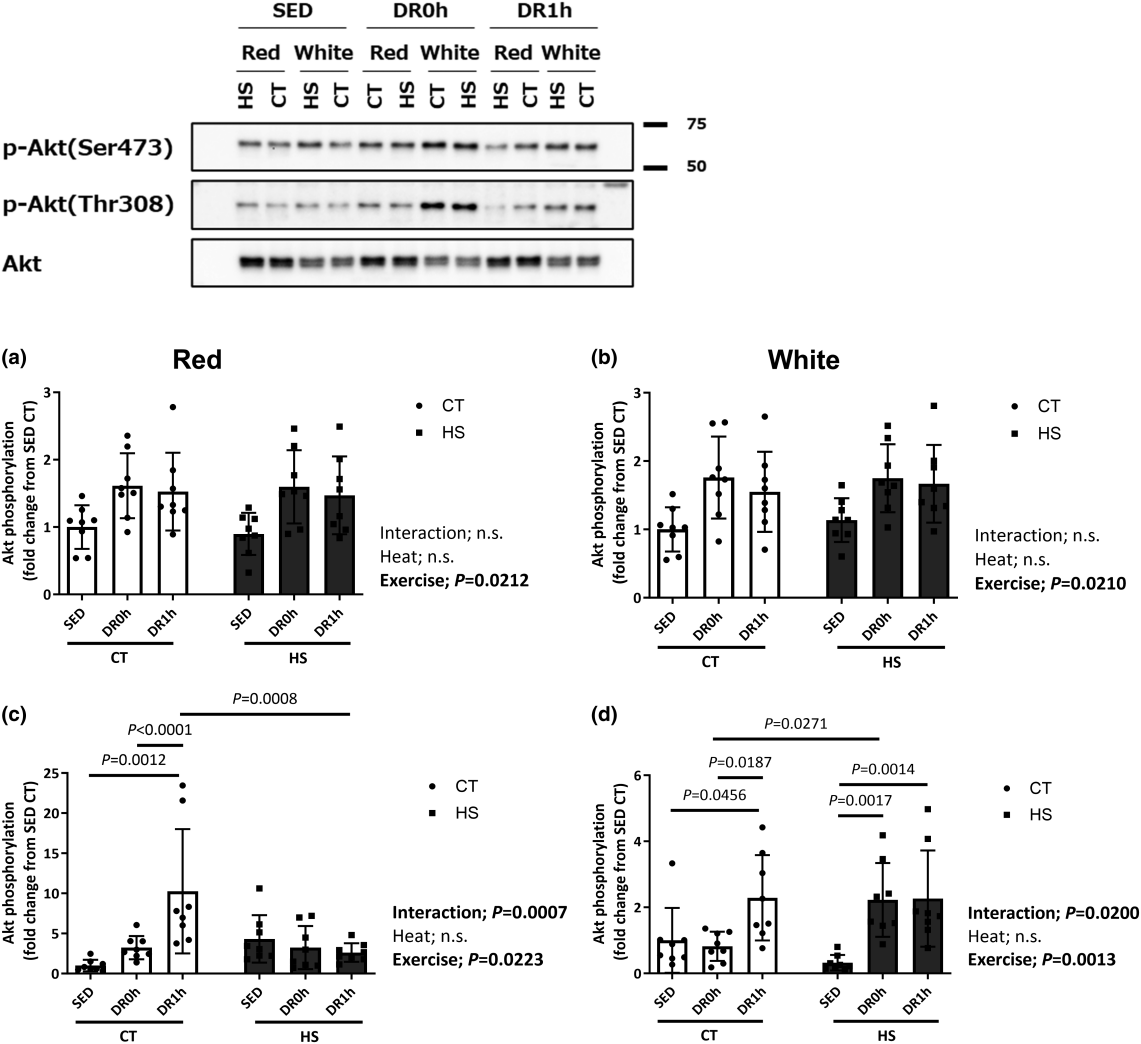
Akt (Ser473 and Thr308) phosphorylation. Phosphorylation ratio of Akt at Ser473 (a, b) and Thr308 (c, d) in the red and white regions of the gastrocnemius muscle following downhill exercise and representative blots. After the detection of phospho‐proteins (Ser473 then Thr308), the blot was reproved for the total protein. CT, control leg; HS, heat‐stressed leg. Samples were collected before exercise (SED) and immediately (DR0h) and 1 h (DR1h) after exercise. The average SED value of the CT leg is presented as 1.0 and the remaining conditions are normalized to this condition. The data are expressed as mean ± standard deviation (SD); *n* = 8 per time point. The results of two‐way ANOVA are displayed.

Figure 6 shows the phosphorylation ratio of Akt (Ser473/Thr308) in the red and white regions of the muscle. The total protein expression of Akt was not significantly different among the experimental groups in both red and white regions of the muscle (data not shown). The level of Akt phosphorylation at Ser473 was higher in DRh1 than in SED in the CT leg. Significant differences in the Ser473 phosphorylation level of Akt were observed after exercise in both red and white regions of the muscle (*p* = 0.0212 and 0.0210; Figure 6a,b). The Thr308 phosphorylation levels changed significantly after downhill exercise in both red and white regions of the muscle (*p* = 0.0007 and 0.0200; Figure 6c,d). The Thr308 phosphorylation level of Akt in the red region after 1 h of the exercise was higher in the CT leg than in the HS leg. The Thr308 phosphorylation level of Akt in the CT legs of both DR0h and DR1h groups was higher than that in the CT leg of the SED group (Figure 6c). In contrast, in the white region, the Thr308 phosphorylation level of Akt was higher in the HS leg than in the CT leg immediately after exercise. The Thr308 phosphorylation level of Akt in the HS legs of both DR0h and DR1h groups was higher than that in the HS leg of the SED group (Figure 6d).

### 3.7 Forkhead box protein 3a phosphorylation

**FIGURE 7:**
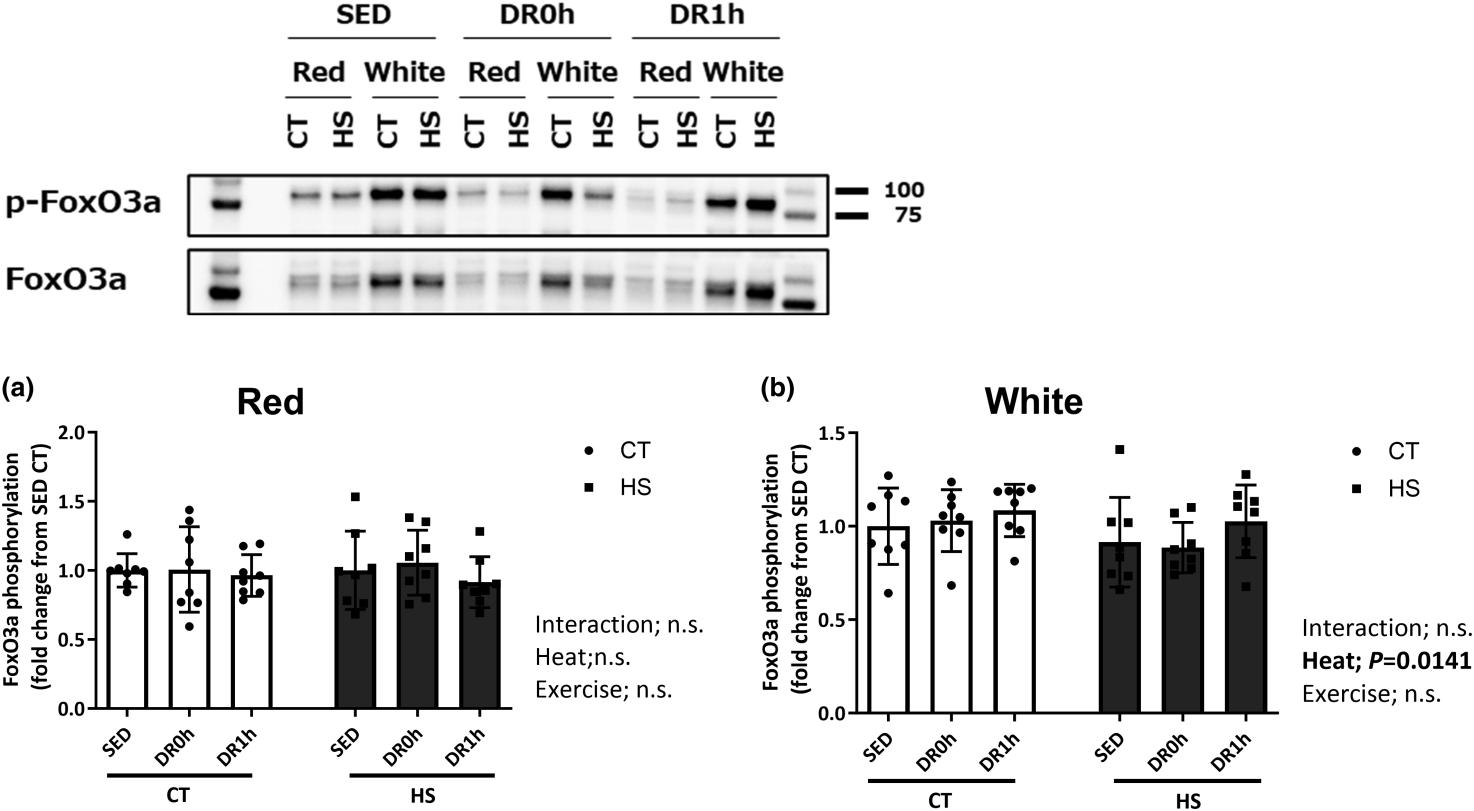
FoxO3a (Ser253) phosphorylation. Phosphorylation level of FoxO3a (Ser253) in the red (a) and white (b) regions of the gastrocnemius muscle following downhill exercise and representative blots. After detection of phospho‐protein, the blot was reproved for the total‐protein. CT, control leg; HS, heat‐stressed leg. Samples were collected before exercise (SED) and immediately (DR0h) and 1 h (DR1h) after exercise. The average SED value of the CT leg is presented as 1.0 and the remaining conditions are normalized to this condition. The data are expressed as mean ± standard deviation (SD); *n* = 8 per time point. The results of two‐way ANOVA are displayed.

Figure 7 shows the phosphorylation levels of Forkhead box protein 3a (FoxO3a) at Ser253 in the red and white regions of the muscle. The total protein expression of FoxO3a was not significantly different among the experimental groups in both red and white regions of the muscles (data not shown). The Ser253 phosphorylation level of FoxO3a in the white region of the muscle was significantly lower in the HS leg than in the CT leg (*p* = 0.0141; Figure 7b), but there was no significant difference between the legs in the red region (Figure 7a).

### 3.8 Puromycin‐conjugated and 4HNE protein expressions

**FIGURE 8:**
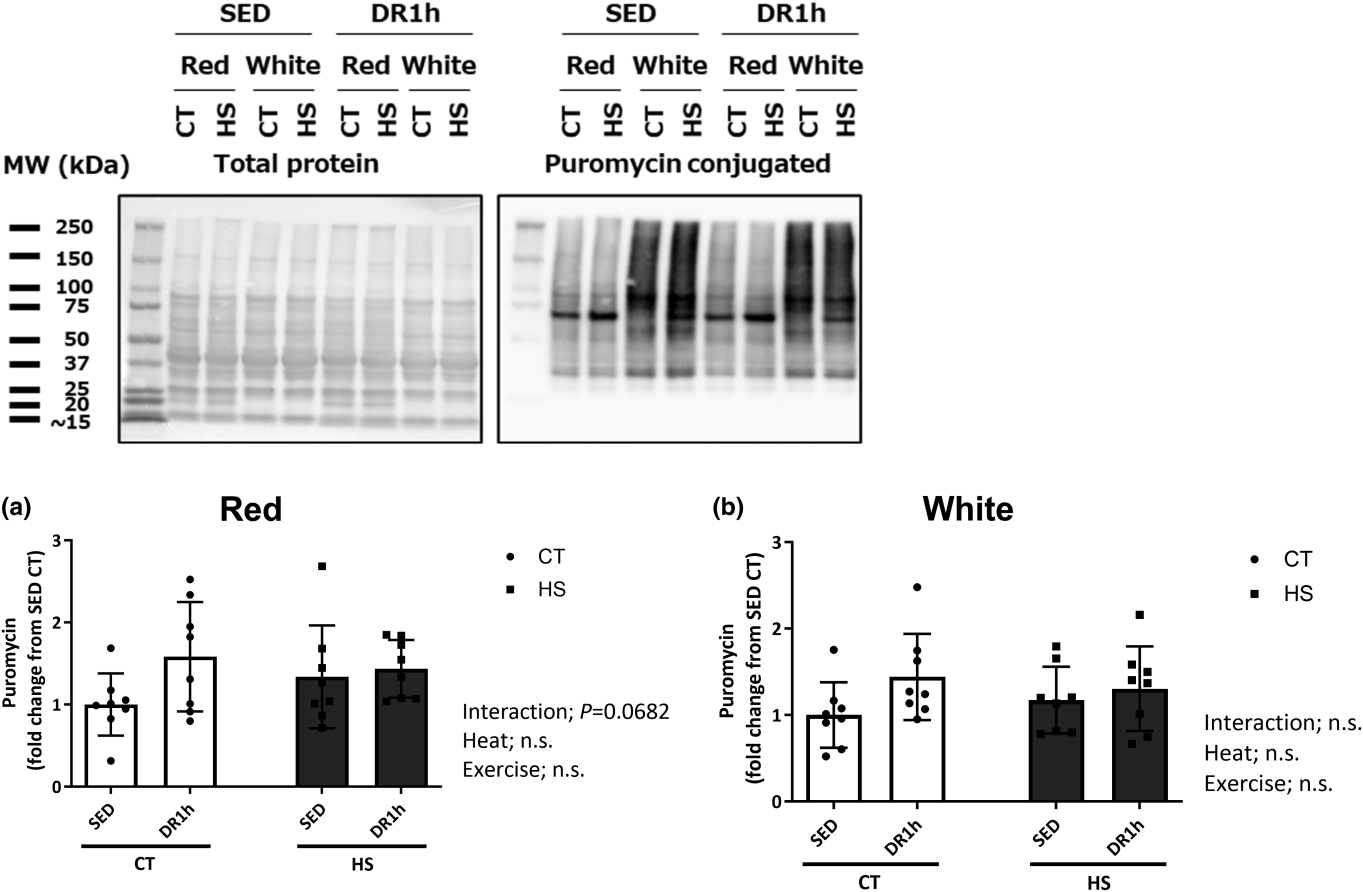
Puromycin‐conjugated and 4HNE protein expression. Expression of puromycin‐conjugated (a, b) and 4HNE (c, d) protein in the red and white regions of the gastrocnemius muscle following downhill exercise and representative blots. After detection of the puromycin‐conjugated protein, the blot was reproved for the 4HNE protein. CT, control leg; HS, heat‐stressed leg. Samples were collected before exercise (SED) and 1 h (DR1h) after exercise. The average SED value of the CT leg is presented as 1.0 and the remaining conditions are normalized to this condition. The data are expressed as mean ± standard deviation (SD); *n* = 8 per time point. The results of two‐way ANOVA are displayed.

Figure 8 shows the expression of puromycin‐conjugated proteins (a and b) and 4HNE (c and d) in the red and white regions of the muscle in a sedentary state and after 1 h of exercise. The puromycin‐conjugated protein level changed significantly after downhill exercise in the red region of the muscles (*p* = 0.0682; Figure 8a), but there was no significant change in the white region (Figure 8b).

Although the 4HNE level significantly increased in response to exercise in the red and white regions of the muscle (*p* = 0.0071 and 0.0565; Figure 8c,d), these differences were unaffected by HS.

### 3.9 Correlation between HSP72 level and intracellular signaling

There was no significant correlation between HSP72 level and singling levels in both red and white regions of the gastrocnemius muscle before (SED, *n* = 8), and immediately (DR0h, *n* = 8) and 1 h (DR1h, *n* = 8) after exercise in both CT and HS legs (Table 1; *p* > 0.05). The linear regression analysis of data of the exercised rats (DR0h and DR1h, *n* = 8, respectively) revealed significant negative correlations between HSP72 level and singling levels in the red region (mTOR, *r* = −0.4163, *p* = 0.0178; Akt (Thr308), *r* = −0.4017, *p* = 0.0227; Table 2). However, no correlation was observed between increased levels (HS leg to CT leg ratio) of HSP72 and intracellular signaling in both red and white regions (Table 2; *p* > 0.05).

**TABLE 1:** Correlations between heat shock protein 72 level and signaling level in both red and white regions of the gastrocnemius muscle.

	*r*	*p*
Heat shock protein 72 level vs. signaling level (red region, *n* = 48)		
mTOR phosphorylation	−0.1423	0.3346
S6K1 phosphorylation	0.0440	0.7664
4E‐BP1 phosphorylation	−0.1170	0.4284
ERK phosphorylation	−0.0697	0.6378
p38 MAPK phosphorylation	0.0241	0.8711
c‐Myc	−0.0619	0.6761
Akt (Ser473) phosphorylation	−0.0443	0.7649
Akt (Thr308) phosphorylation	−0.1620	0.2712
FoxO3a phosphorylation	−0.0857	0.5623
Heat shock protein 72 level vs. signaling level (white region, *n* = 48)		
mTOR phosphorylation	−0.1591	0.2799
S6K1 phosphorylation	0.1513	0.3046
4E‐BP1 phosphorylation	−0.1810	0.2183
ERK phosphorylation	−0.0289	0.8452
p38 MAPK phosphorylation	0.0250	0.8659
c‐Myc	−0.0796	0.5909
Akt (Ser473) phosphorylation	0.1079	0.4653
Akt (Thr308) phosphorylation	0.1455	0.3237
FoxO3a phosphorylation	−0.2133	0.1454

*Note*: Samples were included before (SED, *n* = 8) and immediately (DR0h, *n* = 8) and 1 h (DR1h, *n* = 8) after exercise for each leg (CT and HS legs).

Abbreviations: 4E‐BP1, initiation factor 4E‐binding protein‐1; ERK, extracellular signal regulated kinase; FoxO3a, forkhead box protein 3a; MAPK, mitogen‐activated protein kinase; mTOR, mechanistic target of rapamycin; S6K1, 70‐kDa ribosomal protein S6 kinase 1.

**TABLE 2:** Correlations between the heat shock protein 72 and signaling molecules, as well as their increased levels, in each CT leg in the red and white regions of the gastrocnemius muscle following downhill exercise.

	*r*	*p*
Heat shock protein 72 level vs. signaling level (red region, *n* = 32)		
mTOR phosphorylation	−0.4163	0.0178*
S6K1 phosphorylation	−0.2222	0.2215
4E‐BP1 phosphorylation	−0.2197	0.2269
ERK phosphorylation	−0.0850	0.6438
p38 MAPK phosphorylation	0.1468	0.4226
c‐Myc	−0.2322	0.2010
Akt (Ser473) phosphorylation	−0.2085	0.2521
Akt (Thr308) phosphorylation	−0.4017	0.0227*
FoxO3a phosphorylation	−0.1829	0.3164
Increased heat shock protein 72 level (HS/CT) vs. increased signaling level (HS/CT) (red region, *n* = 16)		
mTOR phosphorylation	0.0741	0.7851
S6K1 phosphorylation	0.2556	0.3394
4E‐BP1 phosphorylation	−0.0592	0.8444
ERK phosphorylation	−0.0534	0.2461
p38 MAPK phosphorylation	−0.1489	0.5821
c‐Myc	0.4082	0.1165
Akt (Ser473) phosphorylation	0.0617	0.8205
Akt (Thr308) phosphorylation	0.3247	0.2198
FoxO3a phosphorylation	−0.2673	0.3168
Heat shock protein 72 level vs. signaling level (white region, *n* = 32)		
mTOR phosphorylation	−0.2399	0.1859
S6K1 phosphorylation	0.1917	0.2932
4E‐BP1 phosphorylation	−0.2419	0.1823
ERK phosphorylation	−0.0473	0.7971
p38 MAPK phosphorylation	0.0260	0.8847
c‐Myc	−0.0862	0.6391
Akt (Ser473) phosphorylation	0.0790	0.6675
Akt (Thr308) phosphorylation	0.3110	0.0832
FoxO3a phosphorylation	−0.2354	0.1946
Increased heat shock protein 72 level (HS/CT) vs. increased signaling level (HS/CT) (white region, *n* = 16)		
mTOR phosphorylation	0.0116	0.9660
S6K1 phosphorylation	0.0331	0.9031
4E‐BP1 phosphorylation	−0.1372	0.6123
ERK phosphorylation	−0.2170	0.4194
p38 MAPK phosphorylation	0.2736	0.3051
c‐Myc	−0.1570	0.5616
Akt (Ser473) phosphorylation	0.4507	0.0797
Akt (Thr308) phosphorylation	0.4144	0.1105
FoxO3a phosphorylation	−0.4353	0.0919

*Note*: Samples were included immediately (DR0h, *n* = 8) and 1 h (DR1h, *n* = 8) after exercise for each leg (CT and HS legs).

Abbreviations: 4E‐BP1, initiation factor 4E‐binding protein‐1; CT, control leg; ERK, extracellular signal regulated kinase; FoxO3a, forkhead box protein 3a; HS, heat‐stressed leg; MAPK, mitogen‐activated protein kinase; mTOR, mechanistic target of rapamycin; S6K1, 70‐kDa ribosomal protein S6 kinase 1.

* *p* < 0.05 Values are indicated as the ratio of the HS leg to CT leg.

## 4 DISCUSSION

In this study, we investigated the effects of HS pretreatment‐induced increase in HSP72 expression on mTOR signaling responses following transient exercise in the rat gastrocnemius muscle. Our results demonstrated that HS‐induced HSP upregulation (1) attenuated mTOR phosphorylation at Ser2448 in the white region of the gastrocnemius muscle, (2) did not adversely affect the downstream signaling of mTOR and protein synthesis following DR in the rat gastrocnemius muscle, and (3) induced differential responses of Akt phosphorylation at Thr308 in the red and the white regions of the gastrocnemius muscle.

One of the main contributions of the study was demonstrating for the first time that the HS pretreatment‐induced increase in HSP72 expression did not adversely affect mTOR downstream signaling, which is related to muscle protein synthesis in response to acute exercise, in the rat gastrocnemius muscle. We revealed that S6K1 and 4E‐BP1 phosphorylation status was similar in both the non‐heated and preheated legs following the downhill exercise. The mTOR signaling pathway plays a central role in muscle protein synthesis (Yoon, 2017). As these signaling responses to DR or resistance exercise are essential for muscle adaptation (Ogasawara et al., 2017), HS pretreatment and increased HSP72 expression are unlikely to prevent skeletal muscle synthesis. In contrast, a previous study demonstrated that prior HS or the consequent accumulation of HSPs inhibits skeletal muscle hypertrophy induced by mechanical overload (Frier & Locke, 2007). It is believed that preconditioning prior to subsequent stressors may elicit protective effects in muscle cells. For instance, HS pretreatment and/or the ensuing HSP72 response may protect against muscle damage induced by acute DR after 48 h and increase the total protein and neonatal myosin heavy chain content following exercise (Touchberry et al., 2012). Moreover, HS pretreatment results in less muscle weight loss and lower ROS production and proteolytic activation than no heat treatment in rats (Naito et al., 2000; Yoshihara et al., 2015). Collectively, these findings demonstrate that HS pretreatment helps to attenuate some signs of muscle injury and atrophy in skeletal muscle (McGorm et al., 2018). As oxidative stress and muscle damage during exercise are required for muscle adaptation (Powers et al., 2020; Vargas‐Mendoza et al., 2021), attenuating these stressors may elicit maladaptive effects on the muscles. However, our findings revealed that HS pretreatment did not suppress S6K1 and 4E‐BP1 phosphorylation with similar levels of 4HNE following DR in the rat gastrocnemius muscle. Moreover, we measured the rate of protein synthesis using the IV‐SUnSET technique. Although we failed to show a significant increase in the protein synthesis rate in both red and white regions of the gastrocnemius muscle, our results demonstrated that downhill exercise increased the rate of protein synthesis by 58% (CT leg) and 43% (HS leg) in the red region and 44% (CT leg) and 30% (HS leg) in the white region. Thus, our findings suggest that increased HSP72 expression does not have a direct effect on mTOR signaling activation and the subsequent protein synthesis.

mTOR phosphorylation in the white region of the gastrocnemius muscle decreased in response to HS pretreatment. Moreover, the linear regression analysis of data of exercised rats revealed a significant negative correlation between HSP72 level and mTOR phosphorylation in the red region. A previous study revealed that HSP72 overexpression inhibits heat shock factor phosphorylation and translation by activating protein phosphatase and inhibiting protein kinase C activity in human epidermoid A‐431 cells (Ding et al., 1998). Another report demonstrated that HSP72 overexpression directly inhibits FoxO3a activation (de‐phosphorylation) at Ser253 in atrophied rat soleus muscle (Senf et al., 2010), suggesting that HSP72 regulates the phosphorylation status of various signaling intermediates (Senf et al., 2008, 2010). However, downstream target (S6K1 and 4E‐BP1) activation and c‐Myc levels were similar between the heat‐stressed and non‐heat‐treated muscles. The transcription factor c‐Myc stimulates protein synthesis, possibly by stimulating ribosome biogenesis (Mori et al., 2021). In addition, c‐Myc overexpression increases ribosome biogenesis and protein synthesis independent of mTOR complex 1 activation in mouse skeletal muscle (Mori et al., 2021). Although HS with HSP72 upregulation is a potential factor for mTOR de‐phosphorylation at Ser2448 in white muscle following DR, these findings suggest that the HS pretreatment‐induced attenuation of mTOR phosphorylation is not associated with protein synthesis stimulated by eccentric exercise.

In the present study, we found region‐specific responses in Thr308 phosphorylation of Akt after DR. In the white region, the Thr308 phosphorylation of Akt was higher in the heat‐stressed leg than in the control leg immediately after the exercise, and the levels of Thr308 phosphorylation of Akt in the HS leg of DR0h and DR1h were higher than those in the HS leg of SED. Akt activation includes phosphorylation of two residues: that of threonine 308 (Thr308) in the activation by the protein kinase PDK1 and that of serine 473 (Ser473) in the hydrophobic motif by the mTORC2 complex (Alessi et al., 1997; Sarbassov et al., 2005; Vanhaesebroeck & Alessi, 2000). We previously demonstrated that acute HS immediately induces the phosphorylation of both Thr308 and Ser473 in the soleus and plantaris muscles in rats (Yoshihara et al., 2016). Activated Akt then phosphorylates its downstream substrates, including glycogen synthase kinase‐3, tuberous sclerosis complex‐2, and FOXO‐family transcription factors (Manning & Cantley, 2007), which induce cell growth, survival, and metabolic signals (Goswami et al., 2005; Hanada et al., 2004). Therefore, our findings suggest that HS pretreatment and increased HSP72 levels affect the downhill exercise‐induced Akt Thr308 phosphorylation and alter muscle adaptation related to cell metabolism. In the red region, the Thr308 phosphorylation level of Akt was higher in the CT leg than in the HS leg 1 h after exercise, and the levels of Thr308 phosphorylation of Akt in the CT leg of DR0h and DR1h were higher than those in the CT leg of SED. Moreover, there was a significant negative correlation between basal HSP72 level and Thr308 phosphorylation level of Akt. In the human skeletal muscle, high‐intensity resistance exercise in a fasted condition inhibits Akt phosphorylation at Thr308 and Ser473 immediately and 24 h after exercise (Deldicque et al., 2008). The downhill exercise condition (16 m/min, −16% incline, 10 min) in the present study primarily recruited slow‐twitch muscle. Downhill exercise at a higher speed (26 m/min, −16% incline, 5 min) further increased the recruitment of red region of the gastrocnemius muscle (red region −14% and white region −10% of glycogen loss). Thus, the intensity and contribution for exercise may be higher in the red slow‐twitch fibers of the muscles than in the white fast‐twitch fibers. Moreover, we discovered that HS lowered FoxO3a phosphorylation in the white region of the muscle regardless of increased Akt phosphorylation at Thr308 and Ser473. However, we could not decipher the significance of this alteration. Therefore, future studies are required to clarify the specific mechanisms that contributed to the HS pretreatment‐induced differential response of Thr308 phosphorylation of Akt in the red and white regions of the muscle following downhill exercise.

The present study was limited by the fact that an endpoint immediately or 1 h after the acute exercise was adopted to demonstrate transient signaling responses to exercise (because the response of most intramuscular signaling pathways to acute exercise is transient in the skeletal muscle of rats (Hayasaka et al., 2014)). Therefore, our study did not consider long‐term muscle adaptation and muscle damage to downhill exercise. Additional research is required to clarify the roles of the identified fiber type specificity in the phosphorylated signaling molecules and pathways in muscle adaptation to downhill exercise. Moreover, the use of a transgenic HSP72‐overexpressing model would provide more insights into the significance of HSP72 in intracellular signaling. However, the present study provides novel and important insights into the differences in HS pretreatment‐induced signaling activation related to protein synthesis in response to eccentric exercise in the gastrocnemius muscle in rats.

## 5 CONCLUSION

Our findings suggest that HS‐induced HSP72 upregulation does not adversely affect the downstream signaling of mTOR and protein synthesis following DR in rat gastrocnemius muscle. Additionally, we found differential responses of Thr308 phosphorylation of Akt between the red and white regions of the gastrocnemius muscle.

## CONFLICT OF INTEREST STATEMENT

No conflicts of interest, financial or otherwise, are declared by the authors.

## ETHICAL APPROVAL

All protocols were approved by the Ethics Committee for Animal Experiment at Sakura Campus, Juntendo University (Approval No. 2021‐31, approved on June 9th, 2021) and followed the principles for the care and use of laboratory animals set by the Physiological Society of Japan.

## Data Availability

All data generated or analyzed during this study are included in this published article.
