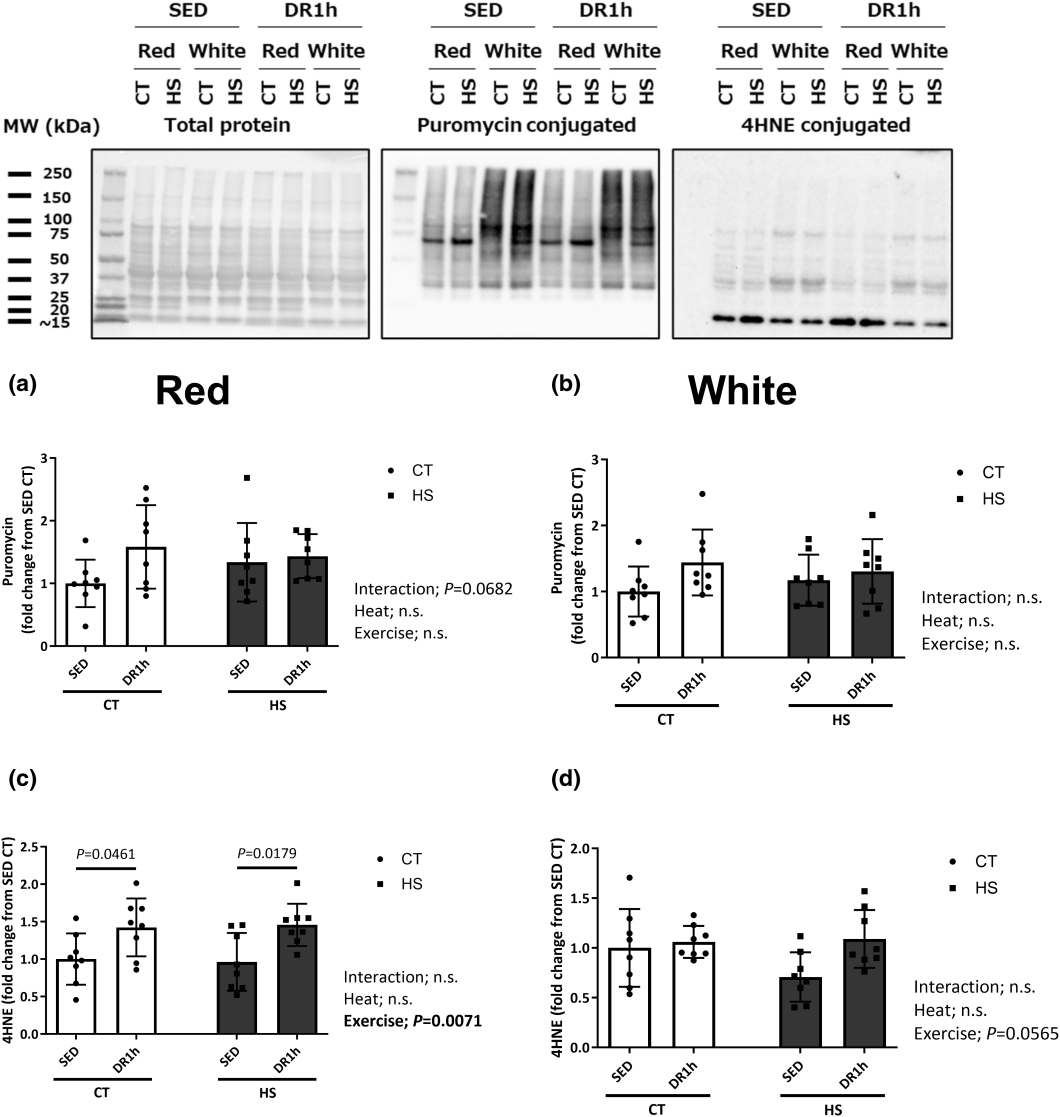# Correction to “Effects of preconditioning with heat stress on acute exercise‐induced intracellular signaling in male rat gastrocnemius muscle”

**DOI:** 10.14814/phy2.70075

**Published:** 2024-10-03

Yoshihara T, Dobashi S, Naito H. Physiol Rep. 2024 Jan;12(1): e15913. doi: 10.14814/phy2.15913.

The published Figure 8 did not include the 4HNE data. The correct version of Figure 8 is provided here. The published legend for Figure 8 matches the correct Figure 8.

This error does not affect the conclusions of the study.

We apologize for this error.